# Predicting the risk of hematoma expansion in acute intracerebral hemorrhage: the GIVE score

**DOI:** 10.1186/s12883-025-04026-6

**Published:** 2025-01-15

**Authors:** Tian-Nan Yang, Xin-Ni Lv, Zi-Jie Wang, Xiao Hu, Li-Bo Zhao, Jing Cheng, Qi Li

**Affiliations:** 1 Department of Neurology, The First Affiliated Hospital of Chongqing Medical University, NO1 Youyi Road, Yuzhong District, Chongqing, 400016 China; 2 Department of Neurology, The Second Affiliated Hospital of Anhui Medical University, Anhui, 230601 China; 3 Department of Neurology, Yongchuan Hospital of Chongqing Medical University, Chongqing, 402160 China; 4 Department of Neurology and Neurosurgery, The Third Affiliated Hospital of Chongqing Medical University, Chongqing, 401120 China

**Keywords:** Intracerebral hemorrhage, Hematoma expansion, Noncontrast computed tomography markers

## Abstract

**Background:**

Numerous noncontrast computed tomography (NCCT) markers have been reported and validated as effective predictors of hematoma expansion (HE). Our objective was to develop and validate a score based on NCCT markers and clinical characteristics to predict risk of HE in acute intracerebral hemorrhage (ICH) patients.

**Methods:**

We prospectively collected spontaneous ICH patients at the First Affiliated Hospital of Chongqing Medical University to form the development cohort (*n* = 395) and at the Third Affiliated Hospital of Chongqing Medical University to establish the validation cohort (*n* = 139). We adopted a revised HE definition, incorporating the standard definition of HE (> 6 mL or > 33%) and intraventricular hemorrhage (IVH) expansion (any new IVH or IVH expansion ≥ 1 ml). The predictive score was formulated based on the parameter estimates derived from the multivariable logistic regression analysis.

**Result:**

The Glasgow Coma Scale, island sign, ventricular hemorrhage and time elapsed from onset to NCCT scan (GIVE) score was created as a total of individual points (0–6) based on Glasgow Coma Scale (2 points for ≤ 11), island sign (1 point for presence), ventricular hemorrhage (1 point for presence), and time elapsed from onset to NCCT scan (2 points for ≤ 2.5 h). The c statistic was 0.72(95% confidence interval [CI], 0.66–0.78) and 0.73(95% CI, 0.63–0.82) in the development and validation cohorts, respectively.

**Conclusion:**

A six-point scoring algorithm has been developed and validated to assess the risk of HE in patients with ICH. This scoring system facilitates the rapid and accurate identification of patients at increased risk for HE.

**Supplementary Information:**

The online version contains supplementary material available at 10.1186/s12883-025-04026-6.

## Introduction

Intracerebral hemorrhage (ICH) is one of the deadliest type of acute stroke, accounting for approximately 10% of the 795,000 strokes in the United States each year, with a subsequent 40% mortality within 1 month [1]. Consequently, individuals surviving ICH commonly face profound cognitive and functional impairments, coupled with an increased risk of future vascular incidents [2, 3]. Approximately one-third of ICH patients presenting within six hours of symptom onset will experience hematoma expansion (HE) which is a key determinant of unfavorable outcomes [4–6]. Several studies suggested that early intensive blood pressure reduction seems to reduce HE and was associated with improved functional outcomes [7, 8].

Recently, several noncontrast computed tomography (NCCT) markers and computed tomographic angiography (CTA) spot signs have been validated to predict HE in ICH patients [9–11]. Various hematoma expansion scores incorporating CTA spot signs have been developed [12, 13]. Nevertheless, considering that CTA is not universally employed in the diagnostic workup for acute ICH patients, NCCT predictors may be an easy to perform alternative to CTA spot signs [14]. Our primary objective was to formulate and validate a predictive score based on NCCT predictors and clinical features to predict hematoma expansion.

## Method

### Study design

We included patients with spontaneous ICH who were admitted to the First Affiliated Hospital of Chongqing Medical University between January 2016 and December 2022 and ICH patients from the Third Affiliated Hospital of Chongqing Medical University between January 2020 and May 2022. The development cohort comprised ICH patients admitted to the First Affiliated Hospital of Chongqing Medical University, while the validation cohort included patients from the Third Affiliated Hospital of Chongqing Medical University. Informed consent was obtained from all patients or their legal representatives. The study protocols were conducted in accordance with the Declaration of Helsinki, and the Ethics Committee approved the study protocol.

Patients meeting the following criteria were included in the study: (1) age > 18 years, (2) diagnosis of spontaneous ICH, (3) absence of anticoagulant treatment, (4) international normalized ratio (INR) < 1.5, and (5) having baseline NCCT within 6 h after symptom onset and follow-up NCCT within 36 h after the baseline scan. Exclusion criteria were as follows: (1) primary intraventricular hemorrhage (IVH), (2) other types of ICH (trauma, tumor, neoplasia, cerebral aneurysm, vascular malformation, hemorrhagic transformation of acute ischemic stroke), (3) underwent surgical treatment (craniotomy or hematoma aspiration), and (4) absence of baseline or follow-up NCCT.

Clinical and demographic data were prospectively collected, including age, sex, history of smoking, history of alcohol consumption, admission systolic and diastolic blood pressures, INRs, Glasgow Coma Scale (GCS), the National Institutes of Health Stroke Scale (NIHSS), medical history of hypertension, medical history of diabetes mellitus, and medical history of previous stroke. GCS and NIHSS scores are used to assess the severity of stroke patients by neurologist in clinical diagnosis and treatment [15–17]. 

### Images acquisition and analysis

**Fig. 1:**
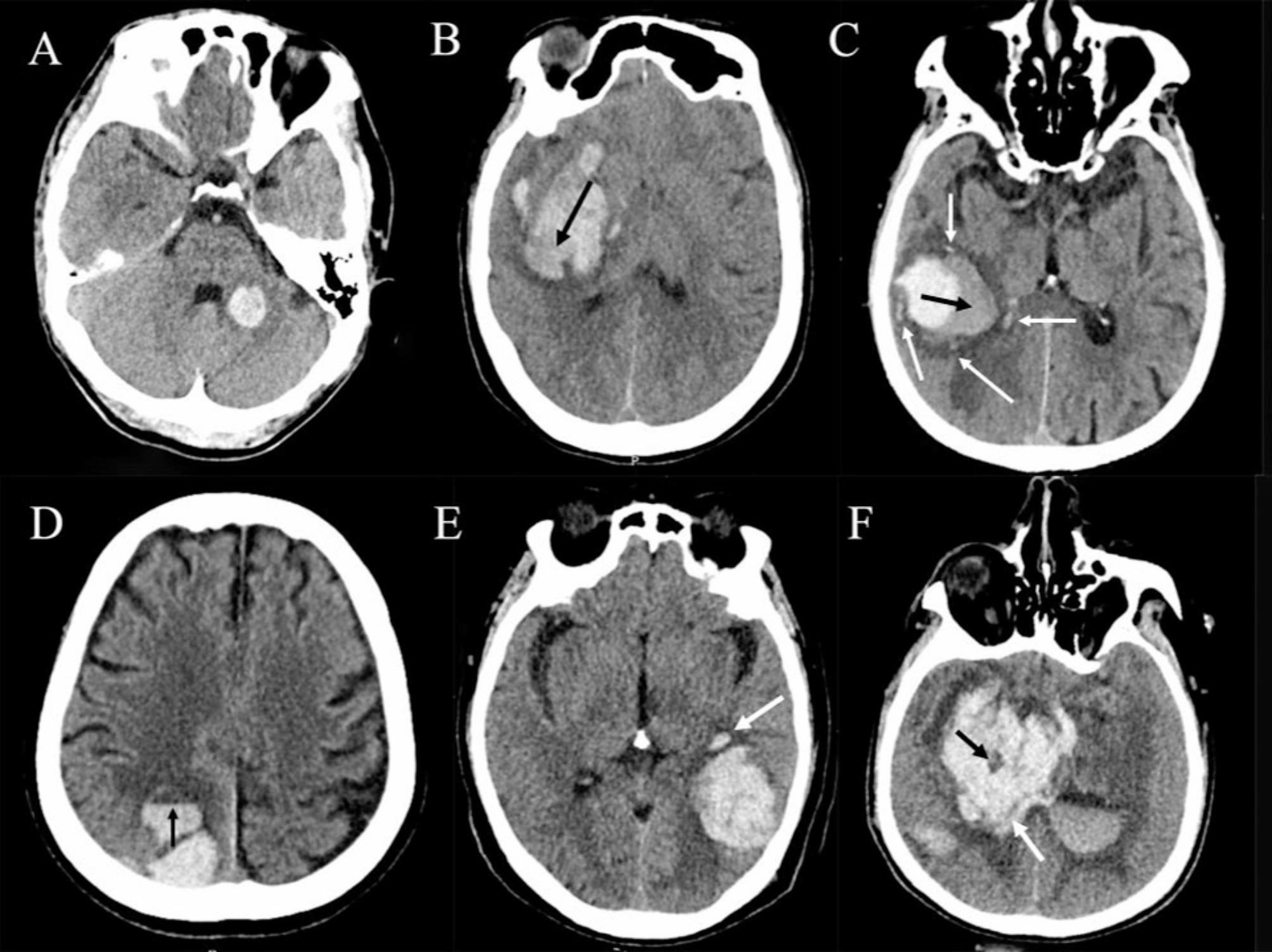
Representative examples of noncontrast computed tomographic markers of intracerebral hemorrhage expansion. (**A**) Regular shape (grade I) and homogeneous density (grade I). (**B**) Irregular shape (grade V) and heterogeneous density (grade V). The arrow indicates swirl sign. (**C**) Island sign (white arrows) and blend sign (black arrow only). (**D**) Fluid level (arrow). (**E**) Satellite sign (arrow). (**F**) Hypodensities (both arrows), swirl sign (both arrows), and black hole sign (black arrow only)

Both the initial and follow-up NCCT scans adhered to established standard clinical protocols. Subsequently, the images obtained were acquired through the picture archiving and communication system and preserved in Digital Imaging and Communications in Medicine (DICOM) format for further review. The volume of hematoma and IVH were measured by a computer-assisted semiautomated planimetric method with the Analyze 12.0 software (Mayo Clinic, Rochester, MN) [18]. Recent studies showed that IVH expansion was independently associated with poor outcome and should be incorporated into the revised hematoma expansion criteria [19, 20]. In our study, HE was defined by using the recent AHA ICH guideline recommendation as meeting at least one of the following four criteria: (1) a relative hematoma growth > 33%, or (2) an absolute hematoma growth > 6 mL, or (3) any new IVH in follow-up NCCT, or (4) IVH expansion ≥ 1 ml [21]. Two experienced neurologists, who were blinded to the patients’ clinical profiles, independently reviewed all images to evaluate the NCCT markers. The readers must determine the presence of the following NCCT markers: black hole signs, island signs, hypodensities, swirl signs, satellite signs, irregular hematoma shapes, heterogeneous hematoma density, and fluid levels. Figure 1 shows an illustrative image of all the NCCT markers described above. In our study, if the shape or density categorical scales ≥ 3, we defined it as irregular hematoma shape or heterogeneous hematoma density [22]. Any discrepancies concerning the identification of these NCCT markers were resolved through a consensus process.

### Statistical analysis

Data analysis was performed using IBM SPSS (version 25.0; IBM Corporation, Armonk, NY). A categorical variable was expressed as a count (percentage), and a continuous variable was expressed as mean with standard deviation (SD), or median with interquartile range (IQR). Comparisons were evaluated through the Mann-Whitney U-test Student t-test, or Chi-square test, as appropriate. In our study, data from the development cohort were utilized to formulate a score for predicting HE. Then, this score was subsequently validated in the other independent cohort. Potential HE predictors, as validated in some clinical trials, were incorporated as candidate variables [5–8, 14]. We preselected following candidate predictor variables: demographics (age, gender), history of hypertension, clinical information (admission systolic and diastolic blood pressures, INRs, GCS, NIHSS, time elapsed from onset to NCCT), NCCT imaging findings (hemorrhage location, hemorrhage volume, presence of IVH, NCCT markers).Cutoffs for continuous variables were estimated using graphical display, and recursive partitioning approach. After univariate logistic regression, only variables with *p* < 0.1 were included into the multivariate logistic regression analysis. The prediction score was created based on the parameter estimates (β coefficients) of the multivariate regression model. Subsequently, the score was validated in both cohorts. The discriminative capacity of the scoring system was assessed by calculating the area under the receiver operating characteristic curve (AUC-ROC). Calibration accuracy was evaluated using the Hosmer-Lemeshow test.

## Result

**Fig. 2:**
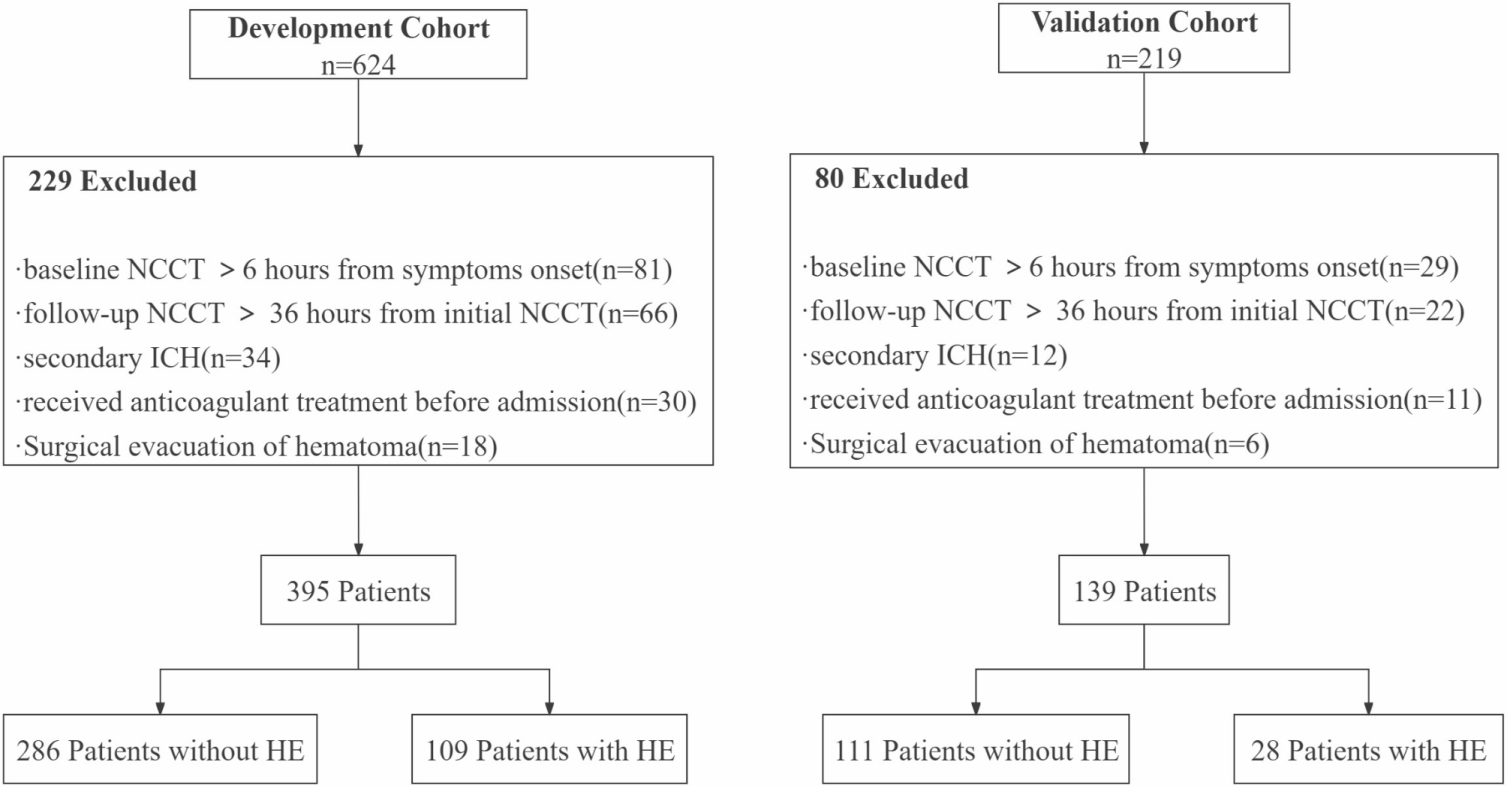
Flowchart of patient selection. HE: hematoma expansion; ICH: intracerebral hemorrhage; NCCT, noncontrast computed tomography

Following the inclusion and exclusion criteria, a total of 534 patients with ICH were enrolled in the study, with 395 in the development cohort and 139 in the validation cohort. The cohort selection process is illustrated in Fig. 2. In development cohort 109 patients (27.6%) experienced HE, while 28 patients (20.1%) developed HE in validation cohort. Table 1 provides a summary of the baseline demographic, clinical characteristics, and NCCT imaging findings for both the development and validation cohorts.

**Table 1:** Characteristics of the development and validation cohorts

Variable	Development Cohort (*n* = 395)	Validation Cohort (*n* = 139)
**Demographic**		
Mean age, years (SD)	59.6(13.4)	62.3(12.5)
Sex, male, n (%) **Medical history**	262(66.3)	92(66.2)
Smoking, n (%)	170(43.0)	53(38.1)
Alcohol consumption, n (%)	122(30.9)	38(27.3)
History of Hypertension, n (%)	287(72.7)	93(66.9)
History of Diabetes mellitus, n (%)	64(16.2)	11(7.9)
History of Stroke, n (%)	77(19.5)	24(17.3)
**Clinical characteristic**		
Admission SBP, mmHg (SD)	174.7(29.2)	167.5(23.2)
Admission DBP, mmHg (SD)	101.4(19.5)	97.8(15.7)
GCS, median (IQR)	14.0(11.0–15.0)	12.0(9.0–14.0)
GCS ≤ 11, n (%)	115(29.1)	58(41.7)
NIHSSS, median (IQR)	10.0(5.0–16.0)	12.0(7.0–19.0)
INR, mean (SD)	1.0(0.1)	1.0(0.1)
Time elapsed from onset to NCCT scan, median (IQR), h	2.2(1.5–3.4)	2.7(2.0-3.6)
Time elapsed from onset to NCCT ≤ 2.5 h, n (%)	219(55.4)	59(42.4)
**NCCT imaging**		
ICH location		
Lobar, n (%)	70(17.7)	17(12.2)
Deep, n (%)	280(70.9)	96(69.1)
Infratentorial, n (%)	45(11.4)	26(18.7)
IVH on baseline NCCT, n (%)	135(34.2)	41(29.5)
Baseline ICH volume, mL (IQR)	11.6(5.8–23.4)	12.5(6.3–25.7)
Hematoma expansion, n (%)	109(27.6)	28(20.1)
Heterogeneous density, n (%)	143(36.2)	80(57.6)
Swirl sign, n (%)	296(74.9)	130(93.5)
Hypodensity, n (%)	227(57.5)	75(54.0)
Black hole sign, n (%)	40(10.1)	31(22.3)
Fluid level, n (%)	23(5.8)	3(2.2)
Irregular shape, n (%)	187(47.3)	104(74.8)
Island sign, n (%)	49(12.4)	15(10.8)
Satellite sign, n (%)	115(29.1)	46(33.1)
90-day mortality, n (%)	56(14.2)	19(13.7)

DBP: diastolic blood pressure; GCS: Glasgow Coma Scale; ICH: intracerebral hemorrhage; INR: international normalized ratio; IQR: interquartile range; IVH: intraventricular hemorrhage; NCCT, noncontrast computed tomography; NIHSS: National Institute of health stroke scale; SBP: systolic blood pressure; SD = standard deviation

### Development cohort characteristics

A univariate analysis was conducted to compare patients with and without HE in the development cohort (Table 2). Patients with HE exhibited a higher NIHSS score (13.0 [7.0–20.5] vs. 10.0 [4.8–15.0], *p* < 0.001), lower GCS score (11.0 [9.0–14.0] vs. 14.0 [12.0–15.0], *p* < 0.001), larger baseline hematoma volume (11.2 ml [5.4–22.4] vs. 13.9 ml [7.0-30.1], *p* = 0.002), a higher incidence of intraventricular hemorrhage on baseline CT (46.8% vs. 29.4%, *p* = 0.001), and a higher rate of mortality at 90 days (27.5% vs. 8.0%, *p* < 0.001). Furthermore, among all NCCT markers in Table 2, in HE patients, except for swirl sign, hypodensity, fluid level, irregular shape and satellite sign, which showed a trend towards significance, other NCCT markers were significant. To evaluate the risk of HE, the admission GCS score and time elapsed from onset to NCCT were dichotomized into GCS ≤ 11 and time elapsed from onset to NCCT ≤ 2.5 h, according to their cutoffs respectively. After taking variables with *p* < 0.1 into the multivariate logistic regression analysis, 4 predictors were found: admission GCS ≤ 11, island sign, IVH on baseline NCCT, and time elapsed from onset to NCCT ≤ 2.5 h. The multivariable logistic regression model is showed in Table 3.

**Table 2:** Univariate analysis comparing ICH patients with and without hematoma expansion

Variable	with Hematoma Expansion *n* = 109 (27.6%)	without Hematoma Expansion *n* = 286 (72.4%)	*P* Value
**Demographic**			
Mean age, years (SD)	60.9(13.7)	59.2(13.2)	0.245
Sex, male, n (%) **Medical history**	78(71.6)	184(64.3)	0.174
Smoking, n (%)	36(33.0)	134(46.9)	0.130
Alcohol consumption, n (%)	31(28.4)	91(31.8)	0.516
History of Hypertension, n (%)	84(77.1)	203(71.0)	0.225
History of Diabetes mellitus, n (%)	24(22.0)	40(14.0)	0.053
History of stroke, n (%)	28(25.7)	49(17.1)	0.055
**Clinical characteristic**			
Admission SBP, mmHg (SD)	176.3(29.4)	174.1(29.1)	0.495
Admission DBP, mmHg (SD)	101.8(22.0)	101.2(18.5)	0.766
GCS, median (IQR)	11.0(9.0–14.0)	14.0(12.0–15.0)	< 0.001
GCS ≤ 11, n (%)	55(50.5)	60(21.0)	< 0.001
NIHSS, median (IQR)	13.0(7.0-20.5)	10.0(4.8–15.0)	< 0.001
INR, mean (SD)	1.0(0.1)	1.0(0.1)	0.198
Time elapsed from onset to NCCT median (IQR), h	2.6(1.6–3.8)	1.7(1.0-2.7)	< 0.001
Time elapsed from onset to NCCT ≤ 2.5 h	80(73.4)	139(48.6)	< 0.001
**NCCT imaging**			
ICH location			0.157
Lobar, n (%)	33(23.9)	91(20.9)	
Deep, n (%)	94(68.1)	292(67.0)	
Infratentorial, n (%)	11(8.0)	53(12.2)	
IVH on baseline NCCT, n (%)	51(46.8)	84(29.4)	0.001
Baseline ICH volume, mL (IQR)	11.2(5.4–22.4)	13.9(7.0-30.1)	0.002
Heterogeneous density, n (%)	48(44.0)	95(33.2)	0.045
Swirl sign, n (%)	88(80.7)	208(72.7)	0.101
Hypodensity, n (%)	70(64.2)	157(54.9)	0.094
Black hole sign, n (%)	17(15.6)	23(8.0)	0.026
Fluid level, n (%)	6(5.5)	17(5.9)	0.868
Irregular shape, n (%)	60(55.0)	127(44.4)	0.058
Island sign, n (%)	23(21.1)	26(9.1)	0.001
Satellite sign, n (%)	38(34.9)	77(26.9)	0.121
90-day mortality, n (%)	38(27.5)	23(8.0)	< 0.001

DBP: diastolic blood pressure; GCS: Glasgow Coma Scale; ICH: intracerebral hemorrhage; INR: international normalized ratio; IQR: interquartile range; IVH: intraventricular hemorrhage; NCCT, noncontrast computed tomography; NIHSS: National Institute of health stroke scale; SBP: systolic blood pressure; SD = standard deviation

**Table 3:** Multivariable logistic regression model from development cohort

Variable	Odds Ratio	95% Confidence Interval	*P* Value
**Multivariate analysis**			
GCS ≤ 11 vs. > 11	2.57	1.52–4.32	<0.001
Island sign presence vs. absence	1.93	1.00-3.73	0.050
IVH on baseline NCCT presence vs. absence	1.67	1.00-2.78	0.048
Time elapsed from onset to NCCT ≤ 2.5 h vs. > 2.5 h	2.32	1.39–3.86	0.001

GCS: Glasgow Coma Scale; IVH: intraventricular hemorrhage; NCCT, noncontrast computed tomography

### Derivation and validation of prediction score

Utilizing the β coefficients obtained from the multivariate logistic regression model, a 4-item prediction score was formulated with a total score ranging from 0 to 6. We chose the first word of the four items and named the prediction score as GIVE score. Meanwhile, Table 4 provided a detailed information about GIVE score. This score underwent validation in both cohorts, with a C statistic of 0.72(95% confidence interval [CI], 0.66–0.78) in the development cohort and 0.73(95% CI, 0.63–0.82) in the validation cohort. The distribution of ICH patients experiencing HE by this predictive score is presented in Table 5. Generally, the proportion of patients experiencing HE increased with higher scores. To maximize clinical usefulness and facilitate the implementation of the score, strata combining individual values were created in the following categories: low (score of 0–2 and incidence rate of 17.3%), medium (score of 3–4 and incidence rate of 36.3%), and high (score of 4–9 and incidence rate of 58.9%) in the development cohort. The score also performed well in the validation cohort. Furthermore, logistic regression analysis was used to ascertain the odds ratio between high risk and low risk, medium risk and low risk in Table 5. The score’s calibration accuracy was evaluated using the Hosmer-Lemeshow test, with *p* > 0.05 in both cohorts.

**Table 4:** Individual components of the GIVE score

Variable	Points
**GCS**	
>11	0
≤ 11	2
**Island Sign**	
Absent	0
Present	1
**IVH on baseline NCCT**	
Absent	0
Present	1
**Time elapsed from onset to NCCT**	
>2.5	0
≤ 2.5	2

GCS: Glasgow Coma Scale; IVH: intraventricular hemorrhage; NCCT, noncontrast computed tomography

**Table 5:** Evaluation hematoma expansion rate with GIVE score

	Hematoma Expansion, *n* (%)	
	Development Cohort	Validation Cohort
C-statistics (95% CI)	0.72(0.66–0.78)	0.73(0.63–0.82)
Score		
0	9/103(8.7)	1/33(3.0)
1	8/40(20.0)	1/13(7.7)
2	26/105(24.8)	7/39(17.9)
3	20/56(35.7)	9/28(32,1)
4	13/35(37.1)	8/19(42.1)
5	24/43(55.8)	2/7(28.6)
6	9/13(69.2)	0/0
Categorized score		
0–2	43/248(17.3)	9/85(10.6)
3–4	33/91(36.3)	17/47(36.2)
5–6	33/56(58.9)	2/7(28.6)
Odds Ratio, (95% CI)		
3–4 vs. 0–2	2.71(1.58–4.65)	4.79(1.92–11.91)
5–6 vs. 0–2	6.84(3.66–12.79)	3.38(0.57–20.02)

CI: confidence interval

## Discussion

We developed and validated a predictive score for assessing the risk of HE by using two large ICH cohorts. The GCS score, island sign, IVH on the NCCT, and time elapsed from onset to NCCT were identified as independent predictors included in our algorithm, resulting in a total score ranging from 0 to 6. Leveraging clinical characteristics and NCCT imaging findings, this score demonstrates the ability to effectively categorize ICH patients into low, medium, or high risk groups for developing hematoma expansion.

Our newly developed GIVE score has clinical implications. The score allows for the stratification of patients into different risk categories based on their likelihood of experiencing HE. This stratification helps clinicians identify ICH patients at low, medium, or high risk, enabling tailored treatment plans and intensified monitoring for those at higher risk. HE is a well-established predictor of unfavorable outcome and was associated with neurological deterioration in ICH patients [23, 24]. Recent evidence suggested that it might be an appealing target for anti-expansion treatment [7, 8]. An exploratory analysis of the Antihypertensive Treatment of Acute Cerebral Hemorrhage 2 (ATACH-2) trial suggested that early (< 2 h) intensive blood pressure reduction may reduce HE and improve functional outcomes in patients with ICH [7]. Furthermore, patients with a bleeding rate > 5 mL/hr were at high risk of HE and might benefit from intensive BP reduction [25]. Therefore, identifying ICH patients at high risk for HE is important for early anti-expansion therapies. To facilitate the timely identification and prevention of HE, patients with a score of ≥ 3, indicating a medium or high risk of HE, should undergo earlier follow-up NCCT scans and receive close observation. This approach aims to enhance the prompt detection of any potential HE, enabling timely intervention and improving patient outcomes.

Several studies have developed and validated predictive scores utilizing various predictors to predict HE. The Brain, BAT Score, and PREDICT A/B Scores were developed based on clinical and neuroimaging characteristics and has shown good accuracy for predicting HE in clinical settings [26–28]. The Brain score incorporates variables such as baseline hematoma volume, recurrent ICH, anticoagulation with Warfarin at onset, intraventricular extension, and time from symptom onset to NCCT, but Brain score does not include any NCCT markers or CTA spot sign. BAT scores include the time from onset to NCCT and two NCCT markers, the blend sign and hypodensity, as predictors. Additionally, admission GCS score, NIHSS score, CTA spot sign, and time between onset to NCCT have been recognized as potential predictors of HE in the PREDICT A/B Scores.

It is noteworthy that all these predictive scores underscore the importance of the time from onset to NCCT. However, among them, only the BAT Score incorporates NCCT markers. Notably, NCCT is more widely available and routinely used in acute ICH diagnostic workups compared to CTA, which is not part of the standard diagnostic process in many institutions. As a result, NCCT markers are easier to obtain than Spot sign in clinical practice. In our study, we also took the GCS and NIHSS scores into consideration, which effectively reflect the severity of ICH in patients, whereas the studies of BAT Score did not involve them. Moreover, compared to the BAT Score, our evaluation of NCCT images included a more comprehensive set of NCCT markers, such as the island sign, black hole sign, swirl sign, and satellite sign, which are not assessed in the BAT Score. Among all types of reported and validated NCCT markers, island sign has a better accuracy than any other kinds of NCCT markers [11]. We involved a broader set of NCCT markers into study, which may improve the accuracy of predictive HE scores and be more suitable in clinical practice. In our study, we developed a prediction score based on the admission GCS score, time elapsed from onset to NCCT, the island sign, and IVH on the NCCT. Compared with previously published prediction HE risks score, our score system is easy to apply, having a total score ranging from 0 to 6 and 4 estimates which can be rapidly evaluated. And we found that the discriminative capability of our score was similar to the performance obtained with BAT Score, but our score performs a more stable discrimination in development and validation cohorts than BAT Score.

In contrast to previous studies, our HE criteria incorporates intraventricular extension. In our study, we adopted a revised HE definition and HE is defined as absolute growth > 6 mL, or relative growth > 33%, or the presence of any new IVH in follow-up NCCT, or IVH expansion ≥ 1 ml. An increasing number of evidence suggested that delayed intraventricular hemorrhage (dIVH) and IVH growth had a negative effect on the functional outcome of ICH patients [20, 29–32]. Furthermore, recent several studies adopted the revised HE criteria and supported an evidence that the revised HE definition had a significantly higher diagnostic accuracy in the prediction of poor functional outcome compared to the conventional definition of HE (> 6 mL or > 33%).^20,31^ Additionally, some studies also demonstrated that NCCT markers could perform well in predicting HE with revised HE definition [31, 32]. This comprehensive definition, particularly the inclusion of intraventricular extension, enhances the predictive value of IVH on baseline NCCT in our scoring system.

The present study has several limitations. Firstly, although the prediction score performed well in both cohorts, further validation in larger multicenter cohorts across different populations or regions is essential to establish its robustness and generalizability. Secondly, the exclusion of patients with a history of warfarin use before symptom onset may limit the applicability of the results to medically managed ICH patients with a history of warfarin use. Third, CTA has higher sensitivity and specificity compared to NCCT in detecting active bleeding [33]. However, our study is limited by the fact that not all patients underwent CTA examination, and therefore, this study focuses solely on NCCT markers. Lastly, our study solely focused on predicting the risk of HE in the early stages of ICH.

## Conclusion

An algorithm for a 6-point scoring system was developed and validated to predict the risk of HE. This system enables the rapid and accurate identification of ICH patients with a high risk of HE, without the need of CTA. It relies on just four indicators derived from clinical and imaging characteristics. Utilizing this score could enhance the efficiency of selecting high risk HE patients in anti-expansion clinical trials. However, future studies are required to validate this prediction score in additional data sets.

## Electronic supplementary material

Below is the link to the electronic supplementary material.

Supplementary Material 1

## Data Availability

The data used and/or analyzed during the current study are available from the corresponding author on reasonable request.

## Abbreviations

CTA: Computed tomographic angiography
CI: Confidence interval
dIVH: Delayed Intraventricular hemorrhage
DBP: Diastolic blood pressure
GCS: Glasgow coma scale
HE: Hematoma expansion
INR: International normalized ratio
IQR: Interquartile range
ICH: Intracerebral hemorrhage
IVH: Intraventricular hemorrhage
NIHSS: National institutes of health stroke scale
NCCT: Noncontrast computed tomography
OR: Odds ratio
SBP: Systolic blood pressure
SD: Standard deviation
