# Supplementary material for: Predicting the risk of hematoma expansion in acute intracerebral hemorrhage: the GIVE score
Source: BMC Neurol. 2025 Jan 15;25:21. doi: 10.1186/s12883-025-04026-6 (PMC11734401; doi:10.1186/s12883-025-04026-6)
Supplement: Supplementary file 1 — Supplementary Material 1 [file 12883_2025_4026_MOESM1_ESM.docx]

Supplement Material

Figure 1: ROC curve of GCS


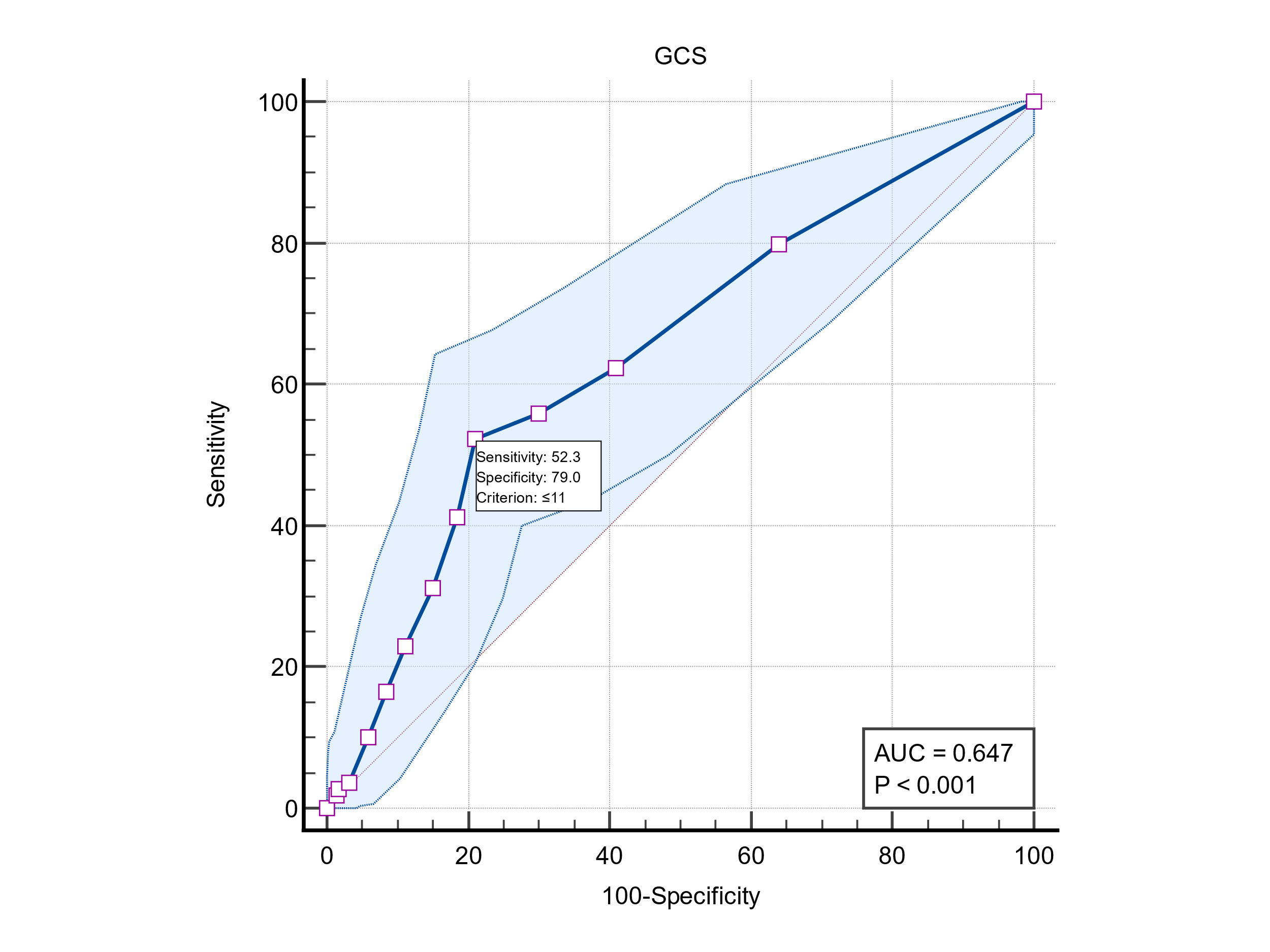


GCS: Glasgow Coma Scale; AUC: Area under Curve

Figure 2: ROC curve of Time elapse from onset to NCCT


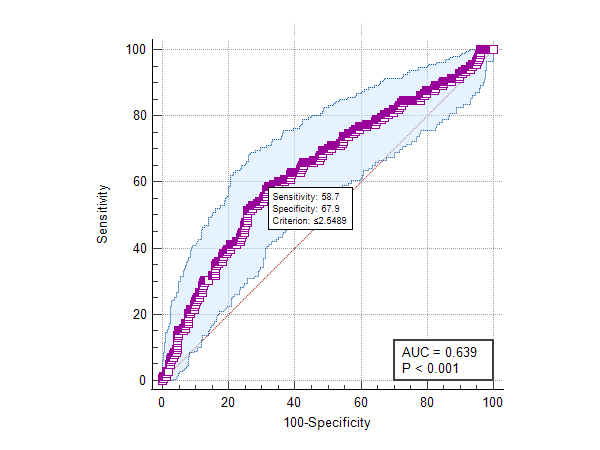


AUC: Area under Curve
